# Observation of the natural course of type 3 spinal muscular atrophy: data from the polish registry of spinal muscular atrophy

**DOI:** 10.1186/s13023-021-01771-y

**Published:** 2021-03-24

**Authors:** Anna Lusakowska, Maria Jedrzejowska, Anna Kaminska, Katarzyna Janiszewska, Przemysław Grochowski, Janusz Zimowski, Janusz Sierdzinski, Anna Kostera-Pruszczyk

**Affiliations:** 1 Department of Neurology, European Reference Network EURO-NMD, Medical University of Warsaw, Warsaw, Poland; 2 Rare Diseases Research Platform, Mossakowski Medical Research Institute, Polish Academy of Sciences, Warsaw, Poland; 3 Student Research Group of Department of Neurology, Medical University of Warsaw, Warsaw, Poland; 4 Department of Genetics, Institute of Psychiatry and Neurology, Warsaw, Poland; 5 Department of Medical Informatics and Telemedicine, Medical University of Warsaw, Warsaw, Poland

**Keywords:** Spinal muscular atrophy (SMA), Neuromuscular disease, Registry, TREAT-NMD, *SMN2* copy number, Type 3 SMA

## Abstract

**Background:**

Spinal muscular atrophy (SMA) is one of the most frequent and severe genetic diseases leading to premature death or severe motor disability. New therapies have been developed in recent years that change the natural history of the disease. The aim of this study is to describe patients included in the Polish Registry of SMA, with a focus on the course of type 3 SMA (SMA3) before the availability of disease-modifying treatments.

**Results:**

790 patients with SMA were included in the registry (173 with type 1 [SMA1], 218 with type 2 [SMA2], 393 with SMA3, and six with type 4 SMA [SMA4]), most (52%) of whom were adults. Data on *SMN2* gene copy number were available for 672 (85%) patients. The mean age of onset was 5 months for SMA1, 11.5 months for SMA2, and 4.5 years for SMA3. In patients with SMA3, the first symptoms occurred earlier in those with three copies of *SMN2* than in those with four copies of *SMN2* (3.2 years vs. 6.7 years). The age of onset of SMA3 was younger in girls than in boys (3.1 years vs. 5.7 years), with no new cases observed in women older than 16 years. Male patients outnumbered female patients, especially among patients with SMA3b (49 female vs. 85 male patients) and among patients with SMA3 with four copies of *SMN2* (30 female vs. 69 male patients). 44% of patients with SMA3 were still able to walk; in those who were not still able to walk, the mean age of immobilization was 14.0 years. Patients with SMA3a (age of onset < 3 years) and three copies of *SMN2* had significantly worse prognosis for remaining ambulant than patients with SMA3b (age of onset ≥ 3 years) and four copies of *SMN2*.

**Conclusions:**

The Registry of SMA is an effective tool for assessing the disease course in the real world setting. *SMN2* copy number is an important prognostic factor for the age of onset and ambulation in SMA3. Sex and age of disease onset also strongly affect the course of SMA. Data supplied by this study can aid treatment decisions.

**Supplementary Information:**

The online version contains supplementary material available at 10.1186/s13023-021-01771-y.

## Background

Spinal Muscular Atrophy (SMA) is an autosomal recessive disease characterized by symmetrical progressive muscle weakness and atrophy due to degeneration of motor neurons in the anterior horn of the spinal cord, with an incidence of approximately 1 in 8000 live births [1–3]. The phenotype of SMA extends from a severe presentation in childhood, with hypotonia and generalized weakness at birth, to an adult-onset disease with mild symptoms. Historically, based on the age of onset and the best motor function achieved, five types of SMA (SMA0, SMA1, SMA2, SMA3, and SMA4) have been distinguished [4]. Type 3 SMA (SMA3) is divided into SMA3a (first symptoms appear before 3 years of age) and SMA3b (onset after 3 years of age) [5, 6].

SMA is caused by homozygous deletion of exon 7 in the survival motor neuron 1 (*SMN1*) gene [7] or—less frequently—by *SMN1* point mutations on one allele occurring in a compound heterozygous state with deletion on the other allele [8, 9]. An increased *SMN2* copy number alleviates the clinical course of SMA [10]. Patients with SMA1 usually have two copies of *SMN2*, those with SMA2 usually have three copies, and those with SMA3 or SMA4 have three or more copies [10–13].

Registries of patients with rare disorders, such as neuromuscular diseases (NMDs), have an important role in monitoring the course of the disease, defining trial or treatment-ready population. In 2007, the Translational Research in Europe and Treatment of Neuromuscular Diseases (TREAT-NMD) project (http://www.treat-nmd.eu) was initiated as a European Committee-funded Network of Excellence to support translational research in the field of NMDs. An important tool to achieve the aims of the TREAT-NMD project was the creation of a global database of patients in national registries. The structure of the registry and the cross-sectional results of this international collaboration have been reported previously [14]. The Polish Registry of SMA was created in 2010 following TREAT-NMD guidelines within a research project funded by the Polish Ministry of Science and Higher Education (641/N-TREAT/09/2010/0). Since then, data have been collected, curated, and updated at a single neuromuscular referral center.

The aim of this study is to describe the patients included in the Polish Registry of SMA and to analyze the clinical course of SMA3 before disease-modifying treatment became available in Poland.

## Methods

### Data collection and entry method

Data were collected by paper or electronic questionnaires, completed by doctors or patients after signed informed consent was obtained (Ethics Committee approval BK/180/2008). The questionnaire included mandatory and highly recommended items, as proposed by TREAT-NMD in 2007 and extended in 2018. Data comprise information on basic motor functions, spirometry, ventilation status, scoliosis, feeding, participation in clinical trials and other registries, and other detailed data. Genetic confirmation of SMA was mandatory and assessment of *SMN2* copy number was highly recommended. The genetic tests were carried out in several laboratories in Poland. If necessary, patients or genetic laboratories were contacted for data clarification. Patients were encouraged to update their data once per year. The accuracy of the data obtained in the questionnaires and compatibility with diagnosis were verified by curators (neurologists), and data were then entered manually onto a Linux-operated server within the Medical University of Warsaw (MYSQL Database) with an option to transfer to Excel for analysis when needed. The database allows the transmission of encoded pseudonymized data to the TREAT-NMD Global Registry.

### Statistical methods

Data were analyzed using SAS 9.4 statistical software (SAS Institute Inc., 100 SAS Campus Drive, Cary, NC 27513-2414, USA). Continuous variables are expressed as mean (+/− SD) or median (95% CI). Discrete variables are presented as numbers or letters and categorical variables are marked accordingly. Statistical analyses describing interrelationships between examined variables and comparisons between groups of patients were carried out using a Mann–Whitney U test, Chi-squared test (χ^2^), ANOVA Kruskal–Wallis, or Wilcoxon test. Pearson and Spearman correlations were used to demonstrate the interdependence of examined traits. Kaplan–Meier models were estimated to assess the risk of immobilization depending on selected parameters.

A multidimensional logistic regression analysis was also carried out to identify independent factors that influence the risk of immobilization. A *p* value of < 0.05 was considered statistically significant.

## Results

### Patient characteristics

**Fig.1:**
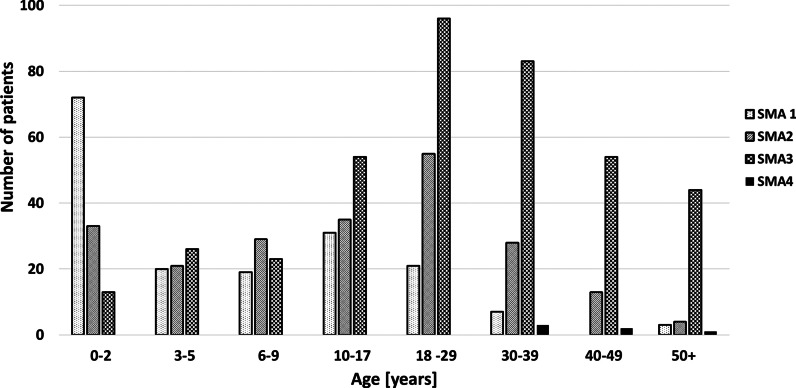
Age distribution of patients by SMA type

In total, 790 patients were included in the registry on 31st October 2019. Enrollment was highest after the registration of nusinersen in Europe and its reimbursement in Poland (January 2019). The registry included 173 patients with SMA1, 218 with SMA2, 393 with SMA3, and six with SMA4. 372 patients were female (female:male = 0.89). The mean age was 8.8 years (SD 11.0) for patients with SMA1, 17.5 years (SD 14.1) for those with SMA2, 28.0 years (SD 16.3) for those with SMA3, and 44.0 years (SD 9.4) for those with SMA4; 52.4% of enrolled patients were adults. The age distribution of all enrolled patients by SMA type is shown in Fig. 1.

For patients with SMA1 and SMA2 the ratio of female to male patients was similar. There was significant male predominance among patients with SMA3b compared with SMA3a (female:male = 0.58 for SMA3b vs. 1.03 for SMA3b; χ^2^ 6.74, *p* < 0.001). All six patients with SMA4 were male.

At the time of data cutoff 11 enrolled patients had died (eight with SMA1, two with SMA2, and one with SMA3).

### Genetic data

In all patients, diagnosis of SMA was confirmed by a genetic test. 98.5% of enrolled patients had homozygous deletion of exon 7 of *SMN1*, and 1.5% had a heterozygous point mutation of the *SMN1* gene coexisting with a deletion. All of these mutations have been previously described elsewhere [8]. Data on *SMN2* copy number were available for 672 (85%) patients. The distribution of *SMN2* copy number by SMA type is presented in Table 1.

**Table 1:** Distribution of *SMN2* copy number by SMA type

Number of *SMN2* copies	SMA1 n = 140 (% of SMA1 patients)	SMA2 n = 182 (% of SMA2 patients)	SMA3 n = 344 (% of SMA3 patients)	SMA4 n = 6 (% of SMA4 patients)
1	2 (1.4%)	1 (0.5%)	0	0
2	53 (38%)	14 (7.7%)	21 (6%)	0
3	80 (57%)	149 (82%)	185 (54%)	1 (17%)
4	5 (3.6%)	18 (10%)	138 (40%)	5 (83%)

Data available for 672 patients

### Age of first symptoms

The mean age of first symptoms (age of onset) was 0.44 years (5 months) for SMA1, 0.96 years (11.5 months) for SMA2, and 4.54 years for SMA3.

**Fig.2:**
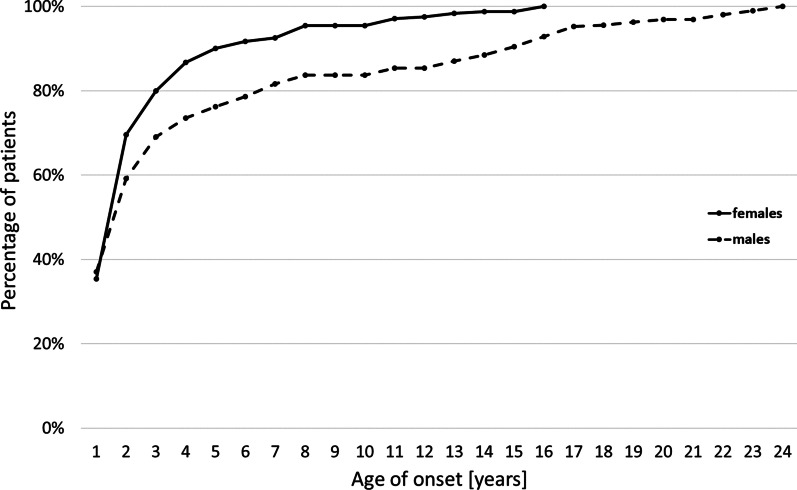
Cumulative data on the age of onset for female and male patients

The age of onset in female patients was significantly lower than in male patients for those with SMA1 (3.5 months [SD 2.28] vs. 6.4 months[SD 10.68], *p* = 0.04) or SMA3 (3.11 years [SD 3.11] vs. 5.67 years [SD 5.64]; *p* < 0.001). Cumulative data on the age of onset showed that the first symptoms occurred by the age of 7 years in 90% of female patients, and by 14 years in 90% of male patients. No new diagnoses were observed in women older than 16 years, whereas the age of onset extended to over 20 years in men (Fig. 2).

59 (15%) of 393 patients with SMA3 had an age of onset under 18 months, with symptom onset occurring between 12 and 17 months in 75% of these patients. The most common first symptoms were walking difficulties (waddling gait) from the beginning. Additionally six other patients with SMA3 reported an age of onset between 1 and 6 months (floppy baby since birth in three cases and “weaker than older sibling or children at the same age” in three cases).

### Influence of *SMN2* copy number on the age of onset of SMA3

Among 250 patients with SMA3 (in the multifactorial analysis), 86 (64%) patients with SMA3a had three copies of *SMN2* and only 38 (28%) had four copies, whereas 47 (40%) patients with SMA3b had three copies of *SMN2* and 61 (52%) had four copies. Only 7% of patients with SMA3 had two copies of *SMN2.*

In patients with SMA3, the first symptoms occurred more than 3 years earlier in patients with three copies of *SMN2* than in those with four copies (mean age 3.01 years vs. 6.71 years; χ^2^ 4.88*, p* < 0.0001) (Table 2).

**Table 2:** Number and age of onset of patients with SMA3 by *SMN2* copy number

SMA3	Number of patients with three copies of *SMN2*	Mean age of onset in patients with three copies of *SMN2*	Number of patients with four copies of *SMN2*	Mean age of onset in patients with four copies of *SMN2*	*p* value
All	133	3.01 years (SD 3.3; 1 month–18 years)	99	6.71 years (SD 5.86; 1 month–20 years)	< 0.0001
Female	72	2.35 years (SD 1.9; 1 month–12 years)	30	5.13 years (SD 4.46; 1 month–16 years)	< 0.0001
Male	61	3.78 years (SD 4.3; 0.5–18 years)	69	7.1 years (SD 5.85 years; 1 month–20 years)	< 0.0001

Data available for 232 patients. Values are expressed as mean (SD; range)

Of the 59 patients with SMA3 with symptom onset before 18 months of age, four (7%) had two copies of *SMN2*, 35 (64%) had three copies, and 16 (27%) had four copies; the *SMN2* copy number was not provided for four patients.

There were significantly fewer female than male patients with four copies of *SMN2* (30 female vs. 69 male patients, female:male = 0.4).

### Ambulation in patients with SMA3

Only nusinersen-naive patients with SMA3 were included in the analysis for ambulation. 172 (44%) of 389 patients with SMA3 were still able to walk. The mean age of loss of ambulation was 14.0 years (SD 11). Female patients lost ambulation earlier than male patients (11.9 years [SD 10.2] vs. 15.7 years [SD 11.4]; *p* = 0.022).

Data obtained from a Kaplan–Meier analysis showed that the probability of preserved ambulation for patients with SMA3 was 80% after a disease duration of 10 years, 68% after 20 years, and 61% after 30 years (Additional file 1: Table S1).

Patients with SMA3a had a significantly worse prognosis for remaining ambulant than those with SMA3b. For patients with SMA3a, the probability of remaining ambulant was 58% at 10 years, 37% at 20 years, and 33% at 30 years compared with 89% at 10 years, 78% at 20 years, and 69% at 30 years for patients with SMA3b (χ^2^ 2.5*, p* = 0.001). This observation was also confirmed when female and male patients were analyzed separately (Additional file 1: Table S1).

*SMN2* copy number was a significant prognostic factor for remaining ambulant. In patients with four copies of *SMN2*, 91% could still walk after 10 years, 82% after 20 years, and 73% after 30 years of the disease, whereas in patients with three copies of the *SMN2* gene, the corresponding percentages were 70%, 60%, and 55% (χ^2^ 4.09, *p* < 0.0001) (Additional file 2: Table S2).

**Fig.3:**
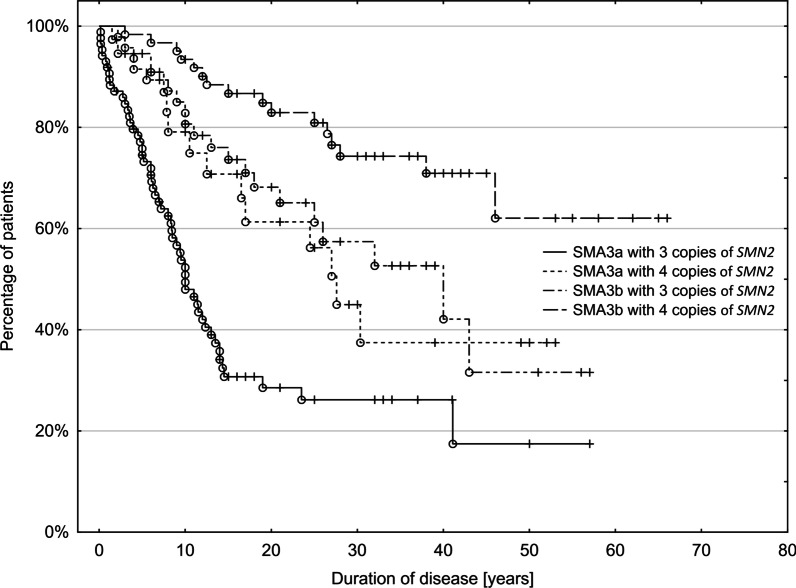
Probability of remaining ambulant with increasing disease duration, by SMA subtype and *SMN2* copy number

There was a significant difference in the probability of remaining ambulant between patients with SMA3a and those with SMA3b for those with three as well as those with four copies of *SMN2* (*p* < 0.05) in all relationships except SMA3a with four copies versus SMA3b with three copies (χ^2^ 0.44, *p* = 0.66). The best prognosis for maintaining the ability to walk was in patients with SMA3b with four copies of *SMN2* (Fig. 3 and Additional file 2: Table S2; duration of disease is the time between onset and immobilization; data available for 232 patients).

The Kaplan–Meier statistics did not show a significant difference between female and male patients in terms of the prognosis for remaining ambulant regardless of the age of onset or *SMN2* copy number.

A multifactorial logistic analysis with step-wise factor selection was carried out on the 250 patients with SMA3, the results of which are presented in Table 3.

**Table 3:** Results of multifactorial logistic analysis with step-wise factor selection

Model fit χ^2^ 53.59, *p* < 0.0001							
Parameter		Estimate	Wald Chi-square	Pr > χ^2^	Odds ratio	95% Wald confidence limits	
Intercept		− 1.37	22.85	< 0.0001			
*SMN2* copies (≤ 3 vs. 4)	1	0.69	5.31	0.021	2.00	0.64	6.74
Sex (male vs. female)	2	0.70	6.25	0.012	2.02	1.16	3.49
Disease duration*	3	0.08	47.69	< 0.0001	1.1	1.06	1.20

*Disease duration = the interval between the onset and the last follow-up

## Discussion

The Polish Registry of SMA was set up under TREAT-NMD initiative in 2010. The main purpose of TREAT-NMD was to improve trial-readiness, advance patient diagnosis and care, and accelerate the search for therapies for patients with NMDs. During the last few decades, the number of SMA registries and registered patients has been growing quickly worldwide. In October 2019, the cut-off date for our analysis, the Polish Registry of SMA included 790 patients, which makes our registry one of the largest national databases of patients with SMA [14, 15]. The most dynamic increase in the number of enrolled patients was directly related to the approval of nusinersen in 2016 in the USA and a reimbursement decision in Poland in December 2018. The first Polish patients began nusinersen treatment in the National Health Program in April 2019. Only 33 patients treated with nusinersen were included in our study (19 with SMA1, ten with SMA2, and four with SMA3) at data cut-off. The four patients with SMA3 were excluded from the analysis on ambulation, so our study was able to characterize Polish patients with SMA3 prior to the era of widely available treatment that changes the natural history of SMA.

Half of our 790 patients had SMA3. SMA1 constituted 22% and SMA2 28% of the population in the registry. The proportion of SMA types in the Polish registry reflects the real-world prevalence of SMA types, although previous data on the incidence of SMA indicate that SMA1 accounts for about 50% of all SMA patients [2]. The predominance of SMA3 has also been observed in other registries [14]. In the Global TREAT-NMD Registry (more than 5000 patients), the proportion of SMA1 patients was even smaller. This situation is probably caused by the severe disease course and high mortality of SMA1 before the era of pharmacotherapy.

Contrary to data in the TREAT-NMD Global Registry or Cure SMA Registry most of our patients (52%) are adults and 37% of them are in the age range 18–39 years [14, 16]. Our observations could therefore provide new insights into the later stages of all SMA types. Additionally, this should raise awareness of the spectrum of issues associated with SMA in adult life, such as education, professional activities, parenthood, or coexisting age-related diseases.

In all 790 patients, the diagnosis of SMA5q was confirmed by genetic testing, which has been widely available in Poland since 2000. In the past decade the *SMN2* copy number has also been assessed routinely at diagnosis. Additionally, many patients who were diagnosed with SMA earlier were referred to genetic laboratories for evaluation of their *SMN2* copy number.

Data on *SMN2* copy number were available in 672 patients in the registry. *SMN2* copy number is known to be an important modifier of the disease, but it is not sufficient to predict phenotype [12, 13]. Nevertheless, three copies of *SMN2* are usually attributed to the SMA2 phenotype [10, 12, 17]. This fact was also confirmed in our study as 82% of patients with SMA2 had three copies of *SMN2*. Interestingly, the percentage of patients with three copies of *SMN2* in our cohort is also high among patients with SMA1 (57%) and SMA3 (54%). In a study of the Spanish population, 86% of patients with SMA1 had two copies of *SMN2* and only 6% had three copies [10]. By contrast, in a Dutch study 21 of 23 patients with a milder course of SMA1 had three copies of *SMN2* [18]. Additionally, in a previous study in the Polish population, 41% of 204 patients with SMA1 had three copies of *SMN2* [13].

Our results are probably related to the structure of the analyzed group, which included a high percentage of patients with SMA1 with a long survival time. This overrepresentation of a milder form of SMA1 in our registry could be explained by the burden on the parents of children with a severe SMA1 phenotype precluding enrollment of acute cases in the registry.

We observed 21 (6%) patients with SMA3 and two copies of *SMN2*. A similar proportion was reported in several studies. Calucho et al. [10] reported that about 5% of patients with SMA3 had two copies of *SMN2* in a combined large cohort of patients. Similarly, in a recently published multicenter study on clinical variability in SMA3, 5% of 182 patients had two copies of *SMN2*. Interestingly, in a group of ambulant patients with SMA3b, this percentage reached 13% [19]. Among German patients with SMA3a, nearly 12% had two copies of *SMN2* [11].

The milder course of the disease in patients with two copies of *SMN2* could be explained by the presence of additional modifying factors, such as the polymorphism c.859G > C in exon 7 or A-44G transition in intron 6 of the *SMN2* gene [20, 21]. We did not test this hypothesis in our cohort. It is recognized that other factors modify the phenotype. In some cases of SMA3 with two copies of *SMN2* or SMA3b with three copies of *SMN2* the polymorphism c.859G > C in exon 7 of the *SMN2* gene was excluded [10, 22].

In our cohort, we observed five patients with SMA1 and four copies of *SMN2*. Single patients with such a discrepancy between genotype and expected phenotype have been reported previously [10, 18]. We are aware that, if in doubt, the *SMN2* copy number should be validated by an expert laboratory with good quality control. This is of utmost importance in pre-symptomatic patients, as it may determine the treatment strategy [23].

44% of patients with SMA3 in our registry were still able to walk. The mean age of immobilization of patients with SMA3 was 14.0 years. In the TREAT-NMD Global Registry, some country-specific differences in the age of immobilization were observed. For example, the mean age of loss of ambulation in Ukraine was 9 years, compared with 19 years in the UK [14]. In a prospective, multicenter study of 28 patients with SMA3 from France, Belgium, and Germany as well as in a study based on the International SMA Registry, the mean age of loss of ambulation was 12 years [17, 19]. Besides being based on a smaller number of patients, both studies included only young adults, compared to the wide age range in our registry.

The Kaplan–Meier survival curves showed that the prognosis for being ambulant was significantly worse for patients with SMA3a than for those with SMA3b. 58% of patients with SMA3a were still able to walk after a disease duration of 10 years, 37% after 20 years, and 33% after 30 years, whereas the prognosis was much better in patients with SMA3b and the corresponding numbers were 89%, 78%, and 69%. A similar analysis was presented in a collaborative study on German and Polish patient data collected from the 1960s to the 1990s. In that study, the probability of being ambulatory was 70% after 10 years, 33% after 20 years, and 22% after 30 years for patients with SMA3a, and the corresponding numbers for SMA3b were 96%, 84%, and 70% [5]. Our data confirm the observation that earlier symptom onset leads to a more severe phenotype [5, 22]. This further supports the need for early diagnosis and pharmacotherapy [23].

We also analyzed the influence of *SMN*2 copy number on the age of onset and walking functions in patients with SMA3. The age of first symptoms in patients with three copies of *SMN2* was more than two times earlier than in those with four copies (3.01 years vs. 6.71 years). Additionally, patients with SMA3a more frequently had three than four copies of *SMN2* (64% vs. 28%), in contrast to SMA3b (40% vs. 52%). The empirical subdivision of patients with SMA3 into SMA3a and SMA3b was first supported by genetic testing by Wirth in 2006 [10, 11, 13, 18, 22].

Although, by definition, SMA3 should be diagnosed in children with symptom onset after 18 months of age [4–6], 59 patients with SMA3 in our cohort had their first symptoms earlier. Our observations show that the age of onset in SMA3 overlaps with that of SMA2, illustrating the continuum of clinical severity. Classification based on age of onset with a cut-off of 18 months to separate SMA2 and SMA3 can result in about 15% of patients not fitting any traditionally defined type of SMA. An age of onset below 18 months was also reported in patients with SMA3 in previous studies [22, 24].

We also confirmed that *SMN2* copy number plays a substantial role in the prognosis for ambulation in SMA3. Patients with four copies of *SMN2* had a significantly higher chance of walking independently after a certain disease duration than patients with three copies of *SMN2*. Additionally, patients with SMA3b with three or four copies of *SMN2* had a substantially better prognosis for ambulation. The largest differences in the probability of preserving the ability to walk were noted between patients with SMA3b with four copies of *SMN2* and patients with SMA3a with three copies of *SMN2*. Multifactorial analysis showed that patients with three or fewer copies of *SMN2* have a two times higher risk of immobilization than those with four copies. Although the number of copies of *SMN2* plays an important role in the disease course, there are certainly other SMA-modifying factors [25]. Better prognosis for ambulation in patients with SMA3 with four copies of *SMN2*, regardless of the age of onset, was also reported in other studies [18, 22, 26].

Interestingly, a recent multicenter study on the natural history of SMA3 in a large cohort reported that age, SMA type, and ambulatory status were significantly associated with changes in mean Hammersmith Functional Motor Scale Expanded (HFMSE) score, but that sex and *SMN2* copy number were not [19].

Our study revealed the influence of sex on some aspects of SMA. There was a significant male predominance among patients with SMA3b (female:male = 0.58, *p* < 0.001) as well as in patients with SMA3 with four copies of *SMN2* (female:male = 0.43). In SMA4, all six patients were male. In SMA1 and SMA3, the age of onset was significantly earlier in female patients.

The influence of sex was observed in some previous studies, which reported a low proportion of female patients with symptom onset after 8 years of age [27, 28]. Male predominance in the chronic form of SMA was also described previously in a Spanish cohort and in the International SMA Registry (female:male = 0.7) [19, 29]. In another study on patients with Spanish origin, a significant predominance of male patients was found among those with SMA2 and SMA3 [22].

The small number of female patients with SMA3 and four copies of *SMN2* in our cohort corresponds with the small number of female patients with SMA3b. In our registry, a significantly smaller number of female patients with four copies of *SMN2* may support the view that a higher proportion of female individuals are asymptomatic carriers of biallelic *SMN1* deletions [28, 30, 31].

In our study, Kaplan–Meier statistics did not show statistically significant differences in the prognosis for ambulation between female and male patients with SMA3 or in subgroups with SMA3a or SMA3b. However, in a multifactorial analysis that took into account sex, *SMN2* copy number, and disease duration, the risk of loss of ambulation for a female patient was two times lower than for a male patient. All these observations indicate a possible relationship between sex and the course of SMA, as reported in other studies [13, 19, 22, 27–31].

## Limitations of the study

We are aware of some limitations of our study. The retrospective data collection and the type of registry (in most cases the questionnaires were completed by patients and verified by a neurologist curator) might influence data accuracy. As most of the patients are adults and present the full spectrum of SMA types, data from the early phase of the disease might not be precise. However, care was taken to verify information with available medical documentation. The relatively high proportion of adult patients in the registry could be because the registry was created at a neuromuscular center for adults and older children. Data on *SMN2* copy number were available from the patients’ routine genetic test results, but genetic analysis for known modifying factors such as polymorphism c.859G > C in exon 7 or A-44G transition in *SMN2* was not performed.

## Conclusions

Our registry-based study allowed us to characterize a large population of patients with SMA and describe the clinical course of SMA3 before the availability of pharmacotherapy. We confirmed that *SMN2* copy number is an important prognostic factor for the age of onset and ambulation in SMA3. Our analysis indicated that *SMN2* copy number, age of disease onset, and sex independently affect the disease course, suggesting the existence of other phenotype modifiers. After 10 years of experience, we conclude that this registry can be a true and effective tool for assessing the clinical course of SMA in the real-world setting.

## Supplementary Information

**Additional file 1.** Probability of being able to walk after the indicated disease durations for all patients with SMA3, SMA3a, and SMA3b, and by sex.

**Additional file 2.** Probability of being able to walk after the indicated disease durations disease for patients with SMA3 and three or four copies of SMN2 separately, and by SMA3a (age of onset < 3 years) and SMA3b (age of onset ≥ 3 years).

## Data Availability

The datasets used and/or analyzed in the current study are available from the corresponding author on reasonable request.

## Abbreviations

CI: Confidence interval
HFMSE: Hammersmith functional motor scale expanded
NMD: Neuromuscular disease
SD: Standard deviation
SMA: Spinal muscular atrophy
*SMN1*: Survival motor neuron 1
TREAT-NMD: Translational research in Europe and treatment of neuromuscular diseases
