# Supplementary material for: Observation of the natural course of type 3 spinal muscular atrophy: data from the polish registry of spinal muscular atrophy
Source: Orphanet J Rare Dis. 2021 Mar 24;16:150. doi: 10.1186/s13023-021-01771-y (PMC7992780; doi:10.1186/s13023-021-01771-y)
Supplement: Supplementary file 1 — Additional file 1. Probability of being able to walk after the indicated disease durations for all patients with SMA3, SMA3a, and SMA3b, and by sex. [file 13023_2021_1771_MOESM1_ESM.docx]

|  | Number of patients | Sex | Kaplan-Meier χ^2^ | p value | Probability of being ambulatory after the indicated duration of disease (%) | | | |
| --- | --- | --- | --- | --- | --- | --- | --- | --- |
|  |  |  |  |  | 10 years | 20 years | 30 years | 40 years |
| All patients | 293 | All |  |  | 80 | 68 | 61 | 60 |
|  | 129 | Female | -1.35 | 0.17 | 80 | 70 | 68 | 65 |
|  | 164 | Male |  |  | 80 | 64 | 57 | 55 |
| SMA3a (onset <3 years) | 159 | All |  |  | 58 | 37 | 33 | 31 |
|  | 81 | Female | -1.18 | 0.24 | 59 | 41 | 41 | 41 |
|  | 78 | Male |  |  | 57 | 34 | 23 | 20 |
| SMA3b  (onset ≥3 years) | 134 | All |  |  | 89 | 78 | 69 | 66 |
|  | 49 | Female | -1.34 | 0.18 | 89 | 85 | 78 | 71 |
|  | 85 | Male |  |  | 89 | 73 | 65 | 64 |

Table S1. Probability of being able to walk after the indicated disease durations for all patients with SMA3, SMA3a, and SMA3b, and by sex.

Duration of disease is the time between onset and immobilization

SMA3a (female and male combined data) versus SMA3b (female and male combined data) (χ^2^ 2.5, *p*=0.001).
