# Supplementary material for: Observation of the natural course of type 3 spinal muscular atrophy: data from the polish registry of spinal muscular atrophy
Source: Orphanet J Rare Dis. 2021 Mar 24;16:150. doi: 10.1186/s13023-021-01771-y (PMC7992780; doi:10.1186/s13023-021-01771-y)
Supplement: Supplementary file 2 — Additional file 2. Probability of being able to walk after the indicated disease durations disease for patients with SMA3 and three or four copies of SMN2 separately, and by SMA3a (age of onset < 3 years) and SMA3b (age of onset ≥ 3 years). [file 13023_2021_1771_MOESM2_ESM.docx]

| Number  of *SMN2* copies | Age of onset  (years) | Probability of being ambulatory after the indicated duration of disease (%) | | | |
| --- | --- | --- | --- | --- | --- |
|  |  | 10 years | 20 years | 30 years | 40 years |
| 3 | All patients | 70 | 60 | 55 | 53 |
|  | <3 | 50 | 28 | 26 | 26 |
|  | ≥3 | 80 | 68 | 57 | 42 |
| 4 | All patients | 91 | 82 | 73 | 71 |
|  | <3 | 80 | 61 | 37 | 37 |
|  | ≥3 | 94 | 83 | 74 | 71 |

Table S2. Probability of being able to walk after the indicated disease durations disease for patients with SMA3 and three or four copies of *SMN2* separately, and by SMA3a (age of onset <3 years) and SMA3b (age of onset ≥3 years). Duration of disease is the time between onset and immobilization

Data available for 232 patients.

SMA3 with three copies vs SMA3 with four copies (χ^2^ 4.09, *p*<0.0001)

SMA3a with three copies vs SMA3a with four copies (χ^2^ 2.79, *p*=0.005)

SMA3a with three copies vs SMA3b with three copies (χ^2^ 3.88, *p*=0.001)

SMA3a with three copies vs SMA 3b with four copies (χ^2^ 6.17, *p*<0.0001)

SMA3a with four copies vs SMA3b with three copies (χ^2^ 0.44, *p*=0.66)

SMA3a with four copies vs SMA 3b with four copies (χ^2^ 2.47, *p=*0.013)

SMA3b with three copies vs SMA3b with four copies (χ^2^ 2.39, *p=*0.016)
